# Key factors determination of hyperuricemia and association analysis among patients with breast cancer: results from NHANES data

**DOI:** 10.3389/fnut.2025.1535879

**Published:** 2025-03-26

**Authors:** Ting-ting Meng, Wen-rui Wang, Yan-qing Zheng, Guan-dong Liu

**Affiliations:** Department of Thyroid and Breast Surgery, Qilu Hospital of Shandong University Dezhou Hospital, Dezhou, China

**Keywords:** influencing factors, hyperuricemia, breast cancer, creatinine, NHANES

## Abstract

**Objectives:**

To explore the factors influencing hyperuricemia in breast cancer patients based on the National Health and Nutrition Examination Survey (NHANES) database.

**Methods:**

The univariate and multivariate generalized linear regression were used to screen the influencing factors of hyperuricemia. Logistic and XGBoost algorithms were used to rank the importance of influencing factors. Receiver Operating Characteristic (ROC) curves and Decision Curve Analysis (DCA) curves were used to assess the predictive performance and clinical benefit. Trend analysis, Restricted cubic spline (RCS) analysis, and generalized additive model were used to explore the relationship between key factor and hyperuricemia.

**Results:**

A total of 359 patients with breast cancer were included, of whom 99 patients had hyperuricemia. Among all variables collected, BMI, total calcium, creatinine, hypertension, and gout were found as independent factors of hyperuricemia (all *p* < 0.05). Among them, Both the 2 algorithms indicated that importance of creatinine on hyperuricemia ranked first. Further, BMI and creatinine levels had higher area under the curve than other variables (BMI: 0.626 [95%CI: 0.574–0.685]; creatinine: 0.722 [95%CI: 0.674–0.777]), but prediction performance difference between them was insignificant (P for Delong test = 0.051). DCA next indicated that creatinine achieved better clinical net benefit than BMI. Further, a detailed positive association between creatinine and hyperuricemia was determined (P for trend<0.001), with a linear relationship (P for non-linear = 0.428).

**Conclusion:**

Creatinine was identified as the most important factor of hyperuricemia in breast cancer patients, as it had independent association with hyperuricemia and favorable prediction performance.

## 1 Introduction

Breast cancer is the most common malignant tumor in women worldwide. The recent breast cancer report by International Agency for Research on Cancer concluded that in 2022, 2.3 million women were diagnosed with breast cancer worldwide, with 670,000 succumbing to the disease; and by 2050, new cases and deaths will have increased by 38 and 68%, respectively (1). The causes of breast cancer are not yet fully understood, and known risk factors include genetic predisposition, factors affecting endogenous hormone levels, additional hormone intake, unhealthy lifestyles, obesity, high breast density, and some benign breast diseases (2, 3). Many studies have reported that chronic inflammation and oxidative stress are inextricably linked to the development of breast cancer (4–6).

Uric acid is a vital metabolite in the human body, which has complex physiological roles, including beneficial effects such as antioxidant, anti-inflammatory, and neuroprotective effects, at the same time, may also lead to unfavorable problems. Currently, most of studies focused on the correlation between uric acid and breast cancer risk. For example, Fan et al. conducted a case–control study involving 1,050 patients, revealing a J-shaped relationship between uric acid levels and breast cancer risk, with a significant increase in risk observed at uric acid concentrations exceeding 3.6 mg/dL (7). A study by Chinese researchers demonstrated elevated uric acid levels in breast cancer patients, establishing an independent association between uric acid levels and breast cancer risk (8). The retrospective cohort study also found significant association between gout and subsequent breast cancer (9). However, a meta-analysis suggested a negative association between serum uric acid and breast cancer risk in women (10). It followed that the association between uric acid and breast cancer remains unclear.

Further, the levels of serum uric acid may also correlate with the prognosis of patients with breast cancer. The previous study found a poor prognosis in breast cancer patients with high serum uric acid concentrations (11). Antioxidant effect of uric acid facilitated the scavenging of reactive oxygen species in breast cancer, thereby driving the proliferation of breast cancer cells (12). These researches remind us that serum uric acid is a valuable prognostic indicator and it is necessary to monitor the uric acid levels of breast cancer patients in a timely manner. The accumulation of serum uric acid may cause the appearance of hyperuricemia, which is characterized by elevated blood uric acid concentrations, typically results from either excessive production or impaired excretion of uric acid (13). The prevalence of hyperuricemia in the U.S. population was 20.2% for men and 20.0% for women (14). The study found that 2.51% of breast cancer patients suffered from hyperuricemia (15). In addition, it was found that patients who treated with combination palbociclib/letrozole presented hyperuricemia (16). These knowledges suggests that the occurrence of hyperuricemia in breast cancer patients is a vital health problem, and it is necessary to closely monitor uric acid levels so that we can make timely treatment plan and improve the prognosis of patients.

At present, there was no study reporting the risk factors associated with the hyperuricemia in breast cancer. Therefore, this study aimed to explore the potential risk factors and determine the core indicator associated with the hyperuricemia risk in breast cancer, using the public National Health and Nutrition Examination Survey (NHANES) data. By identifying the risk factors of hyperuricemia in breast cancer, early screening can be carried out for high-risk patients before the occurrence of the disease. In addition, certain chemotherapy drugs may increase the risk of hyperuricemia through nephrotoxicity or metabolic disorders. After identifying these risk factors, safer chemotherapy drugs can be selected or the dosage can be adjusted.

## 2 Methods

### 2.1 Data source and study participants

Patients data came from the NHANES database, which is a population-based cross-sectional survey that aims to collect information about the health and nutrition of American families. When we incorporated the data, we only considered the data from the past 10 years. We believe that the analysis results of the 10-year data are credible. After retrieval, it was found that although there were records of tumor types in the dataset after 2019, there was no data on the age of breast cancer onset. The tumor duration can be obtained by current age and age of breast cancer onset. We think that tumor duration is a valuable indicator in the progression of breast cancer. In addition, the data on gout was missing after 2017. Therefore, after comprehensive consideration, we only choose the data before 2017 (10 years data from 2007 to 2016).

**Figure 1:**
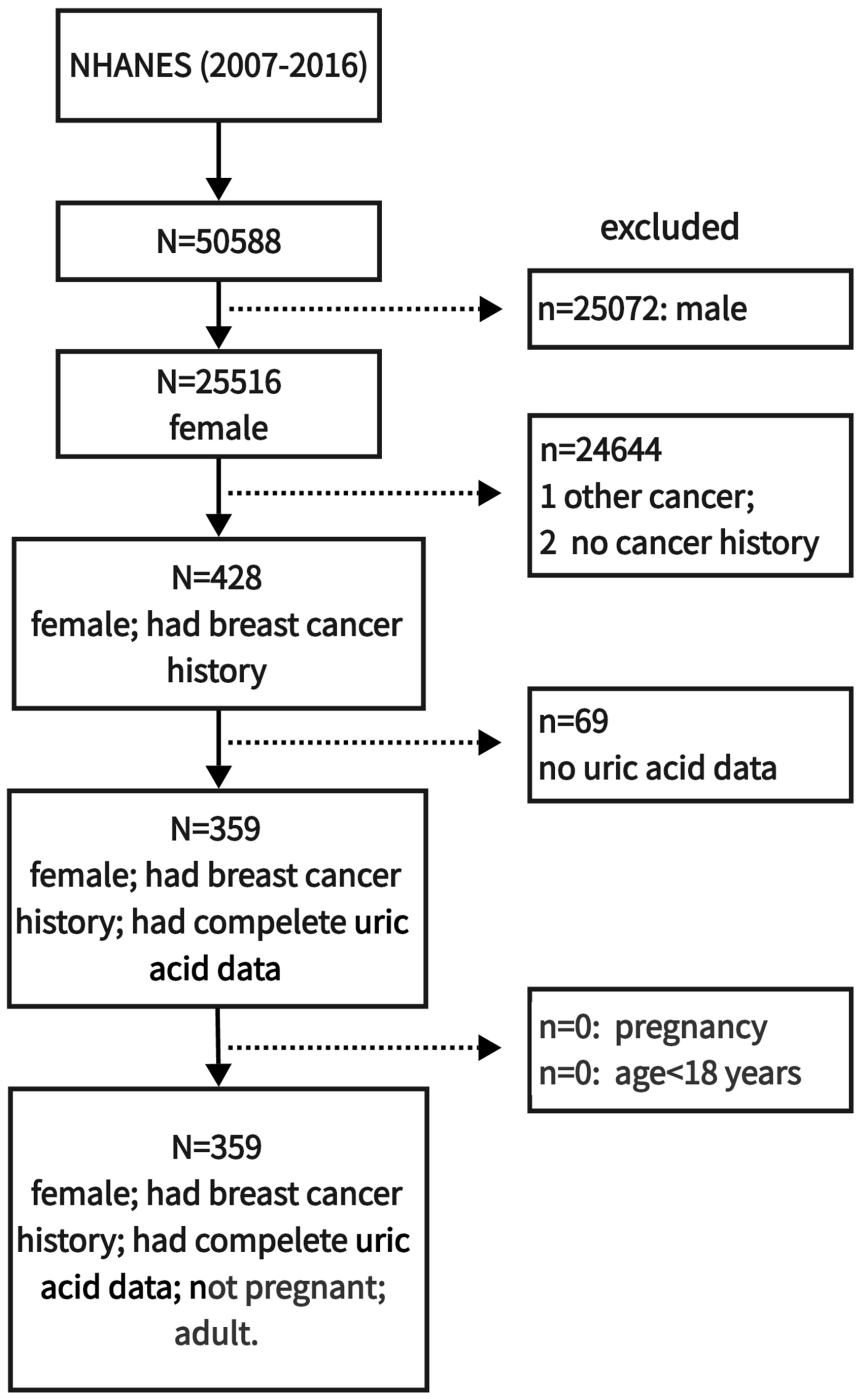
The flow chart of participants selection in this study.

A total of 50,588 participants were collected from NHANES 2007–2016. Then, we selected 428 female breast cancer patients. We found there were only 359 patients with uric acid data in those breast cancer patients. Finally, 359 patients were included. The detailed participants selection was shown in Figure 1.

The self-reported diagnoses for breast cancer were derived from the Medical Conditions Questionnaire (17).

The research design of this study belonged to cross-sectional investigation among observational method from epidemiological prospective.

### 2.2 Indicators and definition

The indicators included in this study include age, poverty index rate (PIR), cotinine (ng/mL), Body Mass Index (BMI), waist circumference (cm), vitamin D (nmol/L), total calcium (mmol/L), phosphorus (mmol/L), sodium (mmol/L), potassium (mmol/L), creatinine (mg/dL), uric acid (mg/dL), white blood cell (WBC) (1,000 cells/μL), lymphocyte (1,000 cells/μL), monocyte (1,000 cells/μL), neutrophils (1,000 cells/μL), age of first diagnosis of breast cancer, course of breast cancer, red blood cell (RBC) (1,000 cells/μL), hemoglobin (g/dL), hematocrit (%), mean corpuscular hemoglobin concentration (MCHC) (g/dL), red cell distribution width (RDW) (million cells/μL), platelet (1,000 cells/μL), race (Hispanic, non-Hispanic), marital status (unmarried, married, other), hypertension (no, yes), diabetes (no, yes), drinking state (never, former, light, moderate, heavy) (18), arthritis (yes, no), gout (yes, no), thyroid diseases (yes, no), liver diseases (yes, no), contraceptive (yes, no), menopause (yes, no).

Hyperuricemia was based on serum uric acid levels, with levels exceeding 7 mg/dL in males and 6 mg/dL, in females classified as hyperuricemia (13).

### 2.3 Statistical analysis

R language was used for data analysis. The quantitative data of normal distribution was described by the mean ± standard deviation, and the ANOVA test was used to compare the two groups. The quantitative data of non-normal distribution was described by the median (P_25_-P_75_), and the Mann–Whitney U test was used to compare the two groups. Qualitative data were described by composition ratio, and the chi-square test was used to compare the two groups. The univariate and multivariate generalized linear regression were used to screen the influencing factors of hyperuricemia. Logistic and XGBoost were used to rank the importance of influencing factors. Receiver Operating Characteristic (ROC) curves and Decision Curve Analysis (DCA) curves were used to assess the predictive performance and clinical benefit of the influencing factors. Trend regression analysis, Restricted cubic spline (RCS) analysis, and generalized additive model were used to explore the relationship between creatinine and hyperuricemia by adjusted for the significant variables with *p* < 0.05 associated with the hyperuricemia in the univariate generalized linear regression. *p* < 0.05 is considered to be statistically significant.

## 3 Results

### 3.1 Patient information

A total of 359 patients with breast cancer were included in this study, of which 99 patients had hyperuricemia. As can be seen from Table 1, patients with hyperuricemia were older, had higher blood levels of cotinine, higher BMI levels, higher total calcium levels, higher creatinine levels, higher uric acid levels, more neutrophils, wider waist circumference, lower hemoglobin, smaller hematocrit, and wider RDW. Besides, there were differences in marital status, hypertension, and gout (all *p* < 0.05). These significant variables were enrolled to next analyses.

**Table 1:** The patients’ information.

Variable		Without hyperuricemia	Hyperuricemia	*P*
Age		68.000 [58.000,75.000]	73.000 [62.000,80.000]	0.002
PIR		2.400 [1.310,4.630]	2.180 [1.180,4.460]	0.381
Cotinine		0.020 [0.010,0.060]	0.030 [0.010,0.240]	0.003
BMI		27.700 [23.980,33.390]	31.000 [27.000,36.200]	<0.001
Waist circumference		97.700 [87.500,107.500]	103.200 [94.800,111.000]	0.003
Vitamin D		76.200 [55.100,98.200]	76.000 [48.000,98.600]	0.711
Total calcium		2.350 [2.280,2.430]	2.380 [2.330,2.450]	0.004
Phosphorus		1.258 ± 0.172	1.254 ± 0.192	0.860
Sodium		140.000 [138.000,141.000]	140.000 [139.000,142.000]	0.199
Potassium		4.000 [3.790,4.200]	4.000 [3.800,4.300]	0.445
Creatinine		0.750 [0.670,0.890]	0.940 [0.760,1.180]	<0.001
Uric acid		4.800 [4.200,5.300]	6.800 [6.300,7.800]	<0.001
WBC		6.600 [5.500,7.900]	6.900 [5.700,8.400]	0.113
Lymphocyte		1.800 [1.400,2.400]	1.800 [1.400,2.200]	0.284
Monocyte		0.500 [0.400,0.700]	0.500 [0.400,0.700]	0.825
Neutrophils		3.900[3.000,5.000]	4.100 [3.300,5.500]	0.043
Age of first diagnosis of breast cancer		54.000 [46.000,63.000]	62.000 [52.000,69.000]	<0.001
Course of breast cancer		9.000 [5.000,16.000]	6.000 [2.000,15.000]	0.013
RBC		4.390 [4.110,4.630]	4.350 [3.980,4.650]	0.373
Hemoglobin		13.400 [12.600,14.100]	13.100 [12.200,13.900]	0.018
Hematocrit		39.700 [37.700,41.300]	38.300 [36.200,41.100]	0.016
MCHC		33.820 ± 0.954	33.754 ± 1.025	0.565
RDW		13.300 [12.700,13.900]	13.600 [12.800,14.300]	0.035
Platelet		235.000 [202.000,275.000]	232.000 [197.000,274.000]	0.674
Race	Hispanic	56 (21.538)	13 (13.131)	0.071
	Non-Hispanic	204 (78.462)	86 (86.869)	
Marital status	Unmarried	28 (10.769)	8 (8.081)	0.030
	Married	138 (53.077)	40 (40.404)	
	Other	94 (36.154)	51 (51.515)	
Hypertension	No	106 (42.400)	14 (14.737)	<0.001
	Yes	144 (57.600)	81 (85.263)	
Diabetes	No	184 (70.769)	61 (61.616)	0.096
	Yes	76 (29.231)	38 (38.384)	
Drinking state	Never	51 (20.732)	25 (26.882)	0.798
	Former	53 (21.545)	20 (21.505)	
	Light	95 (38.618)	32 (34.409)	
	Moderate	34 (13.821)	11 (11.828)	
	Heavy	13 (5.285)	5 (5.376)	
Arthritis	Yes	143 (55.000)	60 (60.606)	0.338
	No	117 (45.000)	39 (39.394)	
Gout	Yes	7 (2.692)	15 (15.152)	<0.001
	No	253 (97.308)	84 (84.848)	
Thyroid diseases	Yes	71 (27.308)	27 (27.273)	0.995
	No	189 (72.692)	72 (72.727)	
Liver diseases	Yes	14 (5.385)	6 (6.061)	0.803
	No	246 (94.615)	93 (93.939)	
Contraceptive	Yes	149 (60.324)	50 (53.763)	0.274
	No	98 (39.676)	43 (46.237)	
Menopause	No	114 (49.565)	38 (42.697)	0.271
	Yes	116 (50.435)	51 (57.303)	

PIR, poverty index rate; BMI, Body Mass Index; WBC, white blood cell; RBC: red blood cell, hematocrit; MCHC, mean corpuscular hemoglobin concentration; RDW. red cell distribution width.

### 3.2 The influencing factors identification of hyperuricemia in breast cancer patients

Next, univariate logistic regression analysis was initially used to explore the association between significant variables and hyperuricemia. However, we found the abnormal odd ratio value for total calcium and creatinine (the excessive wide for 95%CI). Therefore, appropriate generalized linear regression analysis was used to screen the influencing factors of hyperuricemia in breast cancer patients. As shown in Table 2, univariate generalized linear regression found that 11 variables were related to hyperuricemia in breast cancer patients (all *p* < 0.05), including age, BMI, waist circumference, total calcium, creatinine, age of first diagnosis of breast cancer, hemoglobin, hematocrit, RDW, hypertension, and gout, and only these significant variables were entered to the subsequent multivariate regression analysis. However, hemoglobin had high colinearity with other variabels (variance inflation factor = 13.931); hence, hemoglobin was removed and then the colinearity among the remaining variables was non-existent. In multivariate generalized linear regression, we found that 5 indicators including BMI (*β* = 0.021, 95%CI [0.006, 0.036], *p* = 0.006), total calcium (*β* = 0.861, 95%CI [0.451, 1.270], *p* < 0.001), creatinine (*β* = 0.561, 95%CI [0.361, 0.762], *p* < 0.001), hypertension (*β* = 0.107, 95%CI [0.009, 0.205], *p* = 0.033), and gout (*β* = −0.207, 95%CI [−0.403, −0.011], *p* = 0.039) were independently associated with hyperuricemia in breast cancer patients.

**Table 2:** The influencing factors of hyperuricemia in breast cancer patients.

Variable		Univariate		Multivariate	
		β (95%CI)	*P*	β (95%CI)	*P*
Age		0.006 [0.002,0.010]	0.002	−0.002 [−0.008,0.003]	0.470
Cotinine		0.000 [−0.000,0.001]	0.862		
BMI		0.012 [0.005,0.018]	<0.001	0.021 [0.006,0.036]	0.006
Waist circumference		0.005 [0.001,0.008]	0.005	−0.005 [−0.012,0.001]	0.118
Total calcium		0.626 [0.216,1.035]	0.003	0.861 [0.451,1.270]	<0.001
Creatinine		0.723 [0.556,0.891]	<0.001	0.561 [0.361,0.762]	<0.001
Neutrophils		0.026 [−0.001,0.054]	0.060		
Age of first diagnosis of breast cancer		0.007 [0.003,0.010]	<0.001	0.003 [−0.001,0.008]	0.122
Course of breast cancer		−0.002 [−0.006,0.002]	0.326		
Hemoglobin		−0.049 [−0.086,-0.012]	0.010	Seriously collinear	
Hematocrit		−0.017 [−0.030,-0.004]	0.009	−0.006 [−0.019,0.008]	0.418
RDW		0.042 [0.005,0.079]	0.025	0.015 [−0.022,0.053]	0.426
Marital status	Unmarried	Reference			
	Married	0.002 [−0.157,0.162]	0.975		
	Other	0.130 [−0.033,0.292]	0.118		
Hypertension	No	Reference		Reference	
	Yes	0.243 [0.147,0.339]	<0.001	0.107 [0.009,0.205]	0.033
Gout	Yes	Reference		Reference	
	No	−0.433 [−0.621, −0.245]	<0.001	−0.207 [−0.403, −0.011]	0.039

BMI, Body Mass Index; RDW, red cell distribution width.

**Figure 2:**
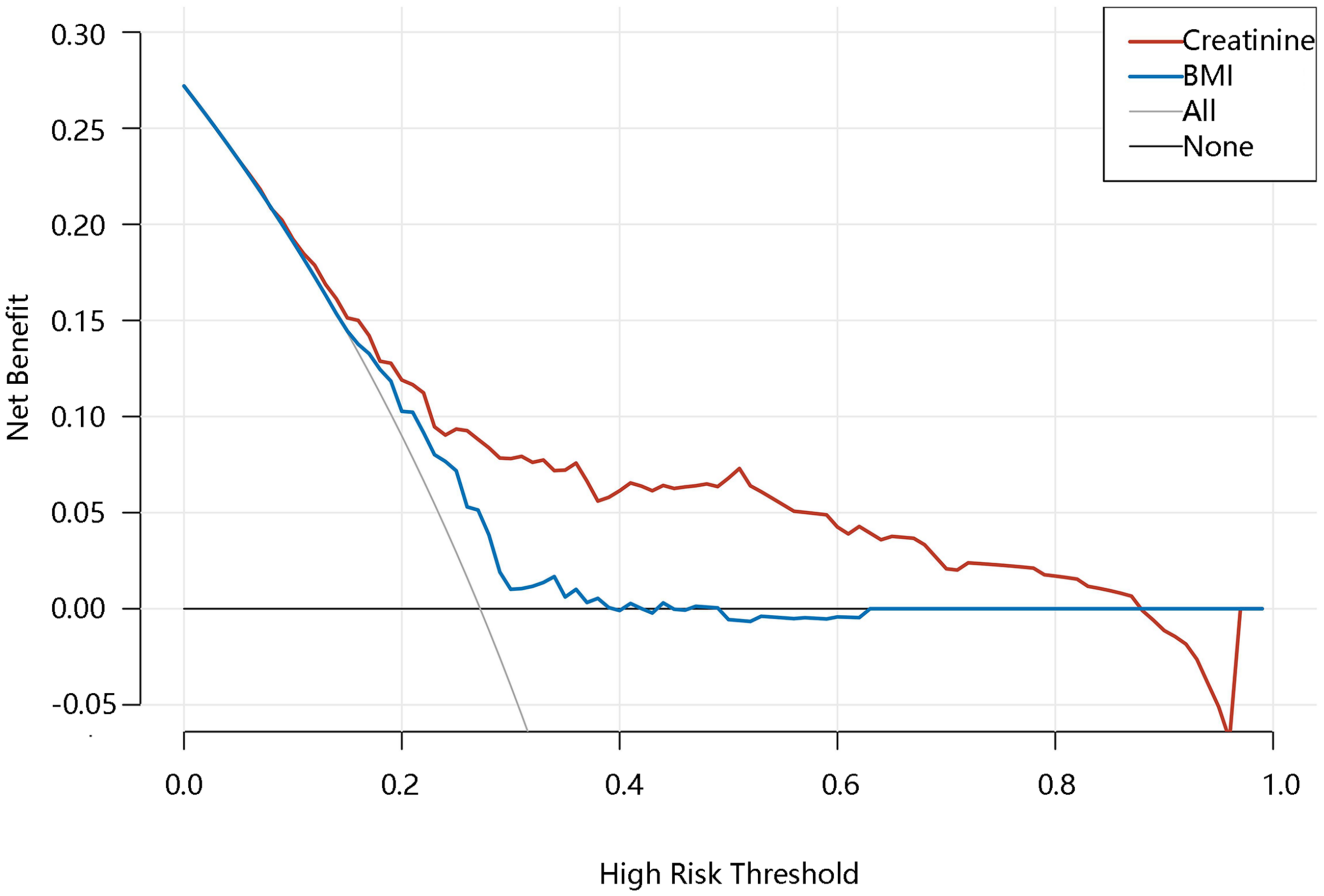
The net benefit of creatinine and BMI. BMI, Body Mass Index.

We further evaluated the importance of the 5 factors on the hyperuricemia by 2 machine learning algorithms. In the logistic algorithms, the importance order was as follows, creatinine>total calcium>gout>hypertension>BMI. In the XGBoost algorithms, the importance order was following: creatinine>BMI > total calcium >hypertension>gout. Both of the two machine learning algorithms showed that the importance of creatinine ranked first among all indicators (Table 3). We further use the ROC curve to evaluate the predictive performance of 5 influencing factors. The Area Under the Curve (AUC) of BMI, total calcium, creatinine, gout, and hypertension predicting hyperuricemia were 0.626 (95%CI = 0.574–0.685), 0.586 (95%CI = 0.525–0.660), 0.722 (95%CI = 0.674–0.777), 0.562 (95%CI = 0.515–0.592), 0.637 (95%CI = 0.586–0.684), respectively (Table 4). It followed that creatinine had the best performance to stratify the hyperuricemia risk. In addition, creatinine had the highest specificity, which contributed to reduce false positive and provided a high level of credibility when excluding diseases. The result of the Delong test (data not shown) showed that creatinine had better prediction performance than total calcium (*p* = 0.006), hypertension (*p* = 0.026), and gout (*p* < 0.001). However, prediction performance between BMI and creatinine had no difference (*p* = 0.051). Therefore, the DCA analysis was further performed to compare their potential value from aspect of clinical net benefit. From Figure 2, when the threshold was 0.15–0.90, the clinical net benefit of creatinine was higher than BMI. Generally speaking, creatinine played the most important role in these five indexes.

**Table 3:** The weight importance of the influencing factors by two machine learning models.

Logistic		XGBoost	
Variable	Weight importance	Variable	Weight importance
Creatinine	0.600	Creatinine	384
Total calcium	0.594	BMI	552
Gout	0.222	Total calcium	254
Hypertension	0.116	Hypertension	27
BMI	0.010	Gout	19

BMI: Body Mass Index.

**Table 4:** Predictive performance of the influencing factors.

	AUC value	Sensitivity	Specificity	Accuracy
BMI	0.626 (0.574–0.685)	0.674 (0.583–0.969)	0.542 (0.219–0.688)	0.735 (0.680–0.765)
Total calcium	0.586 (0.525–0.660)	0.620 (0.506–0.806)	0.578 (0.329–0.675)	0.730 (0.684–0.785)
Creatinine	0.722 (0.674–0.777)	0.489 (0.378–0.840)	0.855 (0.492–0.974)	0.728 (0.582–0.793)
Gout	0.562 (0.515–0.592)	0.972 (0.945–0.987)	0.152 (0.064–0.205)	0.269 (0.220–0.324)
Hypertension	0.637 (0.586–0.684)	0.848 (0.772–0.925)	0.426 (0.380–0.468)	0.534 (0.498–0.571)

BMI, Body Mass Index.

### 3.3 Detailed relationship exploration between creatinine and hyperuricemia

Since we identified creatinine played the most important role in all indexes, we further explored the detailed relationship between creatinine and hyperuricemia. Trend analysis was performed by dividing patients to 4 groups (Q1, Q2, Q3, Q4) according to the quartile of creatinine values. The results (Table 5) showed that the *β* value was gradually increased with the increase of creatinine (Q1 [0.42, 0.69]: reference group; Q2 [0.7, 0.79]: *β* = 0.011, 95%CI [−0.109, 0.131], *p* = 0.857; Q3 [0.8, 0.95]: *β* = 0.053, 95%CI [−0.074, 0.180], *p* = 0.413; Q4 [0.96, 1.93]: *β* = 0.266, 95%CI [0.134, 0.397], *p* < 0.001; P for trend<0.001), suggesting their positive association. In particular, the risk increased significantly when creatinine was greater than 0.96 (*p* < 0.001).

**Table 5:** Trend analysis explored the relationship between creatinine and hyperuricemia.

		Crude model		Adjusted model	
		β (95%CI)	*P*	β (95%CI)	*P*
Creatinine	(0.42, 0.69)	Reference		Reference	
	(0.70, 0.79)	0.054 [−0.067,0.174]	0.383	0.011 [−0.109,0.131]	0.857
	(0.80, 0.95)	0.127 [0.001,0.254]	0.048	0.053 [−0.074,0.180]	0.413
	(0.96, 1.93)	0.401 [0.279,0.524]	<0.001	0.266 [0.134,0.397]	<0.001
	P for trend	0.129 [0.090,0.168]	<0.001	0.083 [0.041,0.125]	<0.001

Adjusting age, BMI, waist circumference, total calcium, age of diagnosis of first breast cancer, hematocrit, RDW, hypertension, gout.

**Figure 3:**
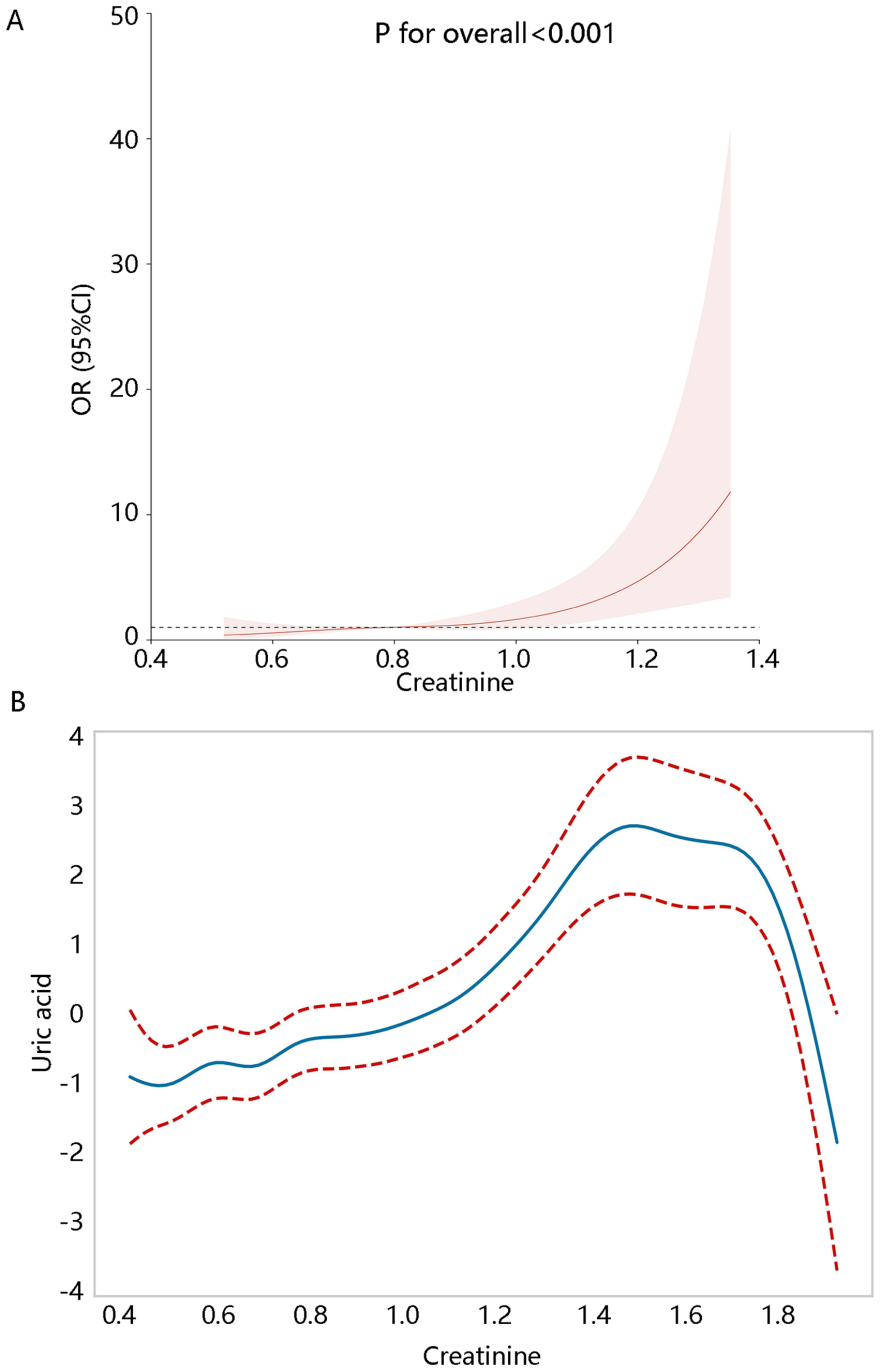
Creatinine levels correlated with hyperuricemia. **(A)** The RCS analysis. **(B)** Generalized additive model (GAM). RCS and GAM analyses all adjusted for the significant variables with *p* < 0.05 associated with the hyperuricemia in the univariate generalized linear regression including age, BMI, waist circumference, total calcium, age of diagnosis of first breast cancer, hematocrit, RDW, hypertension, and gout.

Based on the results of the trend analysis, we further visualized their positive association. RCS analysis (Figure 3A) showed their linear positive relationship (P for overall<0.001, P for non-linear = 0.428) after adjusting for confounding factors. The GAM analysis also showed significant uptrend of uric acid level with the increase of creatinine (Figure 3B). It should be noted that the uric acid level began decreased when creatinine levels were larger than about 1.6, which may affect their positive correlation. We further checked the original data, finding that when creatinine levels were greater than 1.6, all patients (*N* = 13) have hyperuricemia. Considering the small sample size, the descending trend in the latter part of the GAM analysis had limited reference value.

## 4 Discussion

This study is based on the NHANES database to explore the factors affecting hyperuricemia in breast cancer patients. We found that creatinine, total calcium, BMI, hypertension, and gout were the independent factors associated with hyperuricemia; creatinine and BMI had more favorable prediction performance than others. The importance of creatinine on hyperuricemia among them ranked first. Creatinine may be the key indicator associated with hyperuricemia risk.

In this study, we initially found that BMI, total calcium, and hypertension were risk factors. A cross-sectional study based on a Japanese population found that normal-weight central obesity and obesity with central obesity were associated with hyperuricemia (19). A 6-year-long follow-up study of 9,238 patients by Chinese scholars found that patients with hyperuricemia had higher levels of BMI (22.77 vs. 24.68, *p* < 0.001) and that BMI was a risk factor for hyperuricemia (20). Obesity as well as central obesity were found to be risk factors for hyperuricemia in the college population (21). Hyperuricemia caused by obesity may be related to the increase of purine metabolism. Obesity will increase the metabolism of purine in the body, and purine is the precursor of uric acid (22). The acceleration of cell metabolism and the increase in purine intake caused by obesity will increase uric acid production in the body, which will lead to a rise in uric acid levels (23). Regarding the total calcium, a cross-sectional study found a linear relationship between total calcium levels and serum uric acid levels in U.S. adolescents aged 12–19 and an 8% increase in the risk of hyperuricemia for each 0.1 mg/dL increase in total calcium levels (24). A linear relationship between serum calcium and hyperuricemia was found in another study (25), which may involve the mechanisms associated with the inflammation (24). The relationship between hypertension and hyperuricemia has been widely reported, but their causal relationship was uncertain and needed more investigations (26–28).

Creatinine was finally identified as the most important factor associated with hyperuricemia, as it had most favorable prediction performance and achieved better clinical net benefit. Significant positive association between creatinine and hyperuricemia was found, and regulation of creatinine on hyperuricemia needs more investigation. Serum creatinine is a non-enzymatic breakdown metabolite of creatine phosphate in muscle, produced by the body at a fairly constant rate and transported from muscle to the kidneys via circulation (29). It is a commonly used indicator for evaluating renal function and is widely used to calculate glomerular filtration rate, which is easy to monitor. It is found that creatinine level is related to the occurrence and development of many diseases, such as type 2 diabetes mellitus (30) and hypertension (31). In this study, we further found significant association between creatinine and hyperuricemia in breast cancer; but currently, there was no study reporting their association among those patients with breast cancer. Most of the studies only focused on their association (20, 32, 33), but did not limit the research participants. Therefore, future research should take the research participants into consideration when exploring their association, which may exert different findings. We speculated that the relationship between creatinine and hyperuricemia may be related to renal function. When renal function is damaged, its filtration function decreases, resulting in creatinine not being effectively excreted from urine, especially when renal function is seriously damaged, the creatinine level in blood increases significantly. At the same time, the excretion of uric acid is affected, which leads to the increase of uric acid concentration in blood. Obesity and elevated serum uric acid will cause kidney damage or aggravation in different degrees, resulting in decreased excretion of serum uric acid and creatinine (34).

Our study focused on the populations with breast cancer, and the risk factors of breast cancer should not be ignored. Numerous studies have also demonstrated a significant association between uric acid and breast cancer (7, 10, 11, 35–37). A prior research further indicated that elevated uric acid concentrations were predictive of poorer survival outcomes in breast cancer (11). It follows that uric acid is not only involved in the initiation of breast cancer, also it promotes the progression of breast cancer. Current research identified five major risk factors for breast cancer, including age, family history, reproductive factors, estrogen exposure, and lifestyle patterns (38). Given these findings, it is crucial to investigate related factors contributing to breast cancer and its progression, as this may inform more effective clinical management strategies for this population.

Our findings had favorable clinical significance and suggested several future prospects. First, our findings can be applied in the early screening of disease. For breast cancer patients, if an elevated creatinine level is accompanied by hyperuricemia, it may indicate the potential early renal function impairment. This helps in the early detection of renal function abnormalities in breast cancer patients, so as to conduct further examinations and interventions in a timely manner. Second, our findings can provide reference for the treatment decision-making. Many chemotherapeutic drugs need to be excreted through the kidneys, and the renal function status may affect the selection of chemotherapy drug dosage. Therefore, when the early renal function impairment is betokened, it may be necessary to reduce the dosage of certain chemotherapy drugs in order to avoid the accumulation of drugs in the body and produce serious adverse reactions. Third, our findings can be applied in the prognosis assessment. If the patient has a high level of creatinine and hyperuricemia, or continuous deterioration of kidney function (manifested as elevated creatinine and hyperuricemia that are difficult to correct), it may indicate that the tumor has metastasized to distant sites. It is necessary to conduct imaging examinations and tumor marker monitoring more closely.

Our study suggested several future research suggestions at the same time. We can continuously conduct an in-depth study on the molecular mechanisms underlying the co-occurrence of elevated creatinine levels and hyperuricemia in breast cancer patients; and analyze the interaction between breast cancer cells and kidney cells, and determine whether certain cytokines or exosomes secreted by breast cancer cells affect the kidney’s excretion of creatinine and the metabolism of uric acid. In addition, we can perform a large-scale, multicenter, prospective cohort studies to clarify the incidence, development trends, and the dynamic relationship between creatinine levels and hyperuricemia among breast cancer patients; or assess the accuracy of the combined indicators of both in predicting the prognosis of breast cancer patients, and establish a more effective prognostic assessment model.

Finally, our study has some limitations. First, there was one record for uric acid data in the NHANES, while the recommended measurement times of uric acid were two. The uric acid levels may have large fluctuation, which may influence our findings. Second, there may be some confounding factors influencing hyperuricemia that have not been included in this study, such as GLUT-9 and ABCG2. It is uncertain that whether our findings will be affected. Finally, this study belonged to cross-sectional study, and we only clarified the association between creatinine and hyperuricemia. It is not clear whether causality is established.

## 5 Conclusion

Among all related factors analyzed, the importance of creatinine on hyperuricemia ranked first. In addition, creatinine had more favorable prediction performance on hyperuricemia and can achieve better clinical net benefit than others. Creatinine can be regarded as the most important factor of hyperuricemia in breast cancer patients, and their linear positive association was also clarified. Our findings were conducive to the early screening of renal function impairment, provide reference value for the treatment decision-making (especially in the dosage adjustment of chemotherapy drug), as well as distant metastasis assessment.

## Data Availability

The raw data supporting the conclusions of this article will be made available by the authors, without undue reservation.
